# Cold plasma treatment enhances oilseed rape seed germination under drought stress

**DOI:** 10.1038/srep13033

**Published:** 2015-08-12

**Authors:** Li Ling, Li Jiangang, Shen Minchong, Zhang Chunlei, Dong Yuanhua

**Affiliations:** 1 Key Laboratory of Soil Environment and Pollution Remediation, Institute of Soil Science, Chinese Academy of Sciences, Nanjing 210008, China; 2 University of Chinese Academy of Sciences, Beijing 100049, China; 3 Oil Crops Research Institute, Chinese Academy of Agricultural Sciences, Wuhan 430062, China

## Abstract

Effects of cold plasma treatment on seed germination, seedling growth, antioxidant enzymes, lipid peroxidation levels and osmotic-adjustment products of oilseed rape under drought stress were investigated in a drought-sensitive (Zhongshuang 7) and drought-tolerant cultivar (Zhongshuang 11). Results showed that, under drought stress, cold plasma treatment significantly improved the germination rate by 6.25% in Zhongshuang 7, and 4.44% in Zhongshuang 11. Seedling growth characteristics, including shoot and root dry weights, shoot and root lengths, and lateral root number, significantly increased after cold plasma treatment. The apparent contact angle was reduced by 30.38% in Zhongshuang 7 and 16.91% in Zhongshuang 11. Cold plasma treatment markedly raised superoxide dismutase and catalase activities by 17.71% and 16.52% in Zhongshuang 7, and by 13.00% and 13.21% in Zhongshuang 11. Moreover, cold plasma treatment significantly increased the soluble sugar and protein contents, but reduced the malondialdehyde content in seedlings. Our results suggested that cold plasma treatment improved oilseed rape drought tolerance by improving antioxidant enzyme activities, increasing osmotic-adjustment products, and reducing lipid peroxidation, especially in the drought-sensitive cultivar (Zhongshuang 7). Thus, cold plasma treatment can be used in an ameliorative way to improve germination and protect oilseed rape seedlings against damage caused by drought stress.

Oilseed rape (*Brassica napus* L.) is one of the world’s leading economic oilseed crops, and major source of dietary lipid^1^. The production of oilseed rapeseed and rapeseed oil of China has reached to 49 and 13 million metric tons in year 2009, respectively^2^. Drought stress is an important abiotic factor that limits oilseed rape production and yield, accounting for about 30% crop loss^3^,^4^. During drought stress, seed germination is assumed to be hampered, which results in poor crop stand, and inhibition of seedling growth^5^,^6^,^7^,^8^.

Drought stress usually induces the accumulation of reactive oxygen species (ROS), such as superoxide radical, hydrogen peroxide, superoxide and singlet oxygen, which can destroy normal metabolism through oxidative damage to lipids, proteins and nucleic acids^9^. On the other hand, antioxidant enzymes including superoxidase (SOD; EC 1.15.1.1), peroxidase (POD; EC 1.11.1.7) and catalase (CAT; EC 1.11.1.6) become activated to combat ROS^9^,^10^. Osmotic adjustment is another important strategy to deal with drought stress in plants^11^. It has been observed that plants accumulate osmolytes such as soluble sugars and soluble proteins to maintain osmotic equilibrium and the integrity of membranes when they are subjected to drought stress^12^,^13^.

Plasma, known as the fourth state of matter, is composed of excited atoms, molecules, ionized gases, radicals and free electrons. It is widely used in commercial applications ranging from microelectronic technology, medicine, food processing, fusion power, and ion implantation to material modification^14^. Cold plasma is a non-thermal technology, which can be generated under atmospheric and low-pressure conditions, using radio frequency or microwave sources^15^. The cold plasma contains different oxygen radicals, charged particles, ions and UV light, which response for the stimulating effects on seeds^16^. The number of potential applications of cold plasma in agriculture has grown significantly in recent years. Cold plasma treatment is thought to be a fast, economic and pollution-free method to improve seed performance, plant growth and ultimately plant production^17^,^18^. This treatment plays a crucial role in a broad spectrum of plant development and physiological processes in plants, including the promotion of seed germination and seedling growth^19^,^20^, activation of photosynthesis^21^,^22^, regulation of carbon and nitrogen metabolism^23^,^24^. Plasma treatments are thought to enhance the ability of plants to cope with biotic and abiotic stress, such as drought stress^25^ and disease stress^26^. Plasma treatment has been found to promote seedling growth, activities of SOD and POD, increase proline concentration of wheat under drought stress^25^. Jiang *et al*. also reported that cold plasma treatment increase activities of POD, polyphenol oxidase (PPO; EC 1.10.3.1) of tomato under disease stress^26^.

However, little information is available on the evaluation of cold plasma effect on oilseed rape responses under drought stress. The objectives of the current study were (1) to investigate the influences of cold plasma treatment on seed germination, seedling growth, antioxidant enzyme activities and osmolytes content of oilseed rape under drought stress; and (2) to explore how the regulatory mechanisms underlying oilseed rape resistance to drought stress are altered by cold plasma treatment.

## Results

### Seed germination

**Figure 1:**
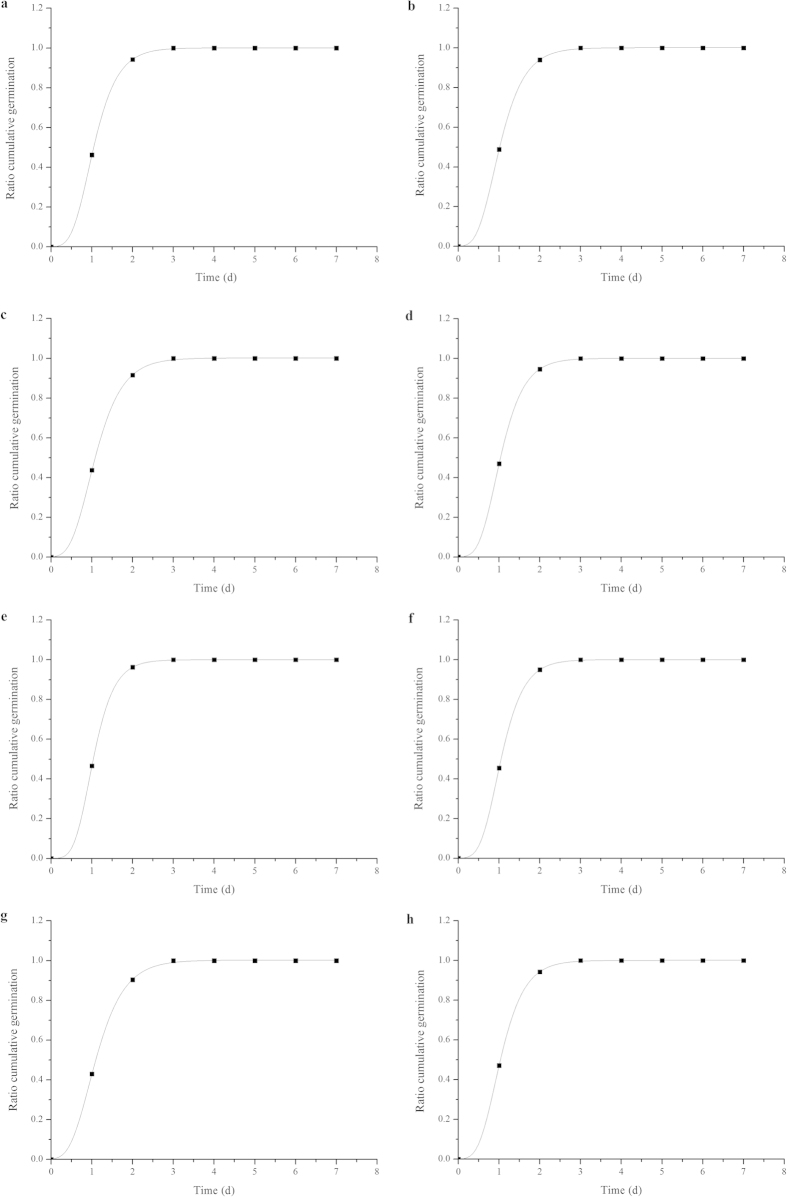
Richards’ function fitted to the germination of WW (**a**), WW + plasma (**b**), DS (**c**) and DS + plasma (**d**) of Zhongshuang 7 and WW (**e**), WW + plasma (**f**), DS (**g**) and DS + plasma (**h**) of Zhongshuang 11 oilseed rape seeds. WW: well watered; WW + Plasma: well watered + plasma; DS: drought stress; DS + Plasma: drought stress + plasma.

Positive influence of cold plasma treatment was recorded on seed germination of Zhongshuang 7 and Zhongshuang 11 under drought stress. Germination improvement was significantly more pronounced in Zhongshuang 7 than in Zhongshuang 11. Under drought stress, the germination rate, germination index and vigor index of Zhongshuang 7 were significantly reduced by 13.98% (*p* < 0.001), 28.14% (*p* < 0.001) and 29.91% (*p* = 0.003), while those of Zhongshuang 11 were significantly reduced by 8.89% (*p* = 0.008), 23.73% (*p* < 0.001) and 4.80%, respectively, compared with the well-watered seeds (Table 1). The cold plasma treatment significantly increased the germination rate, germination index and vigor index by 6.25% (*p* = 0.004), 6.89% (*p* = 0.003) and 29.59% (*p* = 0.007) in Zhongshuang 7 and 4.44% (*p* = 0.015), 4.77% (*p* = 0.013) and 19.64% (*p* = 0.001) in Zhongshuang 11, respectively, compared to the drought-stressed seeds. The germination data was well fitted by the Richards’ function (Fig. 1). The value of *Me* in the plasma treated seeds of both cultivars were lower than those in the non-treated seeds under drought stress, which indicated that the speed of germination was enhanced by the cold plasma treatment under drought stress (Table 2). No significant differences in the parameters *Vi, Qu and Sk* in Zhongshuang 7 and Zhongshuang 11 were found among the non-treated and plasma treated seeds under drought stress.

### Seedling growth

Seedling growth of Zhongshuang 7 and Zhongshuang 11 was significantly inhibited by drought stress (Table 3). Cold plasma treatment markedly improved seedling growth under well-watered and drought stress conditions. The dry weight of shoot and root, length of shoot and root and lateral root number of Zhongshuang 7 treated by cold plasma were significantly increased by 16.67% (*p* = 0.021), 20.22% (*p* = 0.019), 42.72% (*p* = 0.010), 19.09% (*p* = 0.006) and 29.12% (*p* = 0.025), and those of Zhongshuang 11 were improved by 15.00% (*p* = 0.006), 15.16% (*p* = 0.016), 30.09% (*p* = 0.002), 19.83% (*p* = 0.004) and 38.14% (*p* = 0.016), respectively, compared to the drought-stressed seedlings.

### Seed apparent contact angle

**Figure 2:**
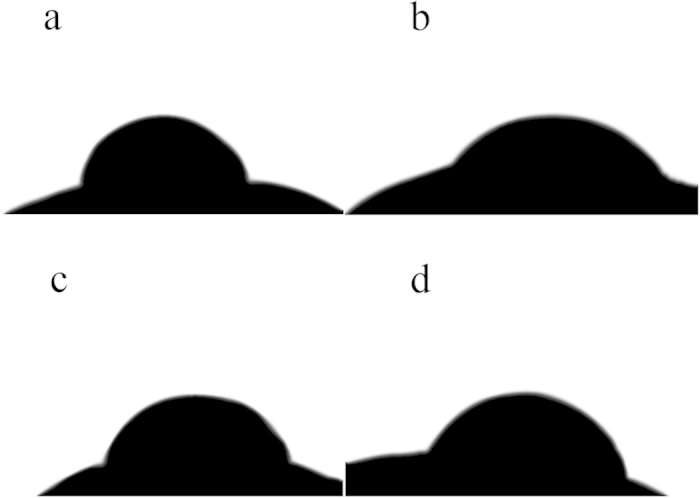
Apparent contact angle of non-treated (**a**) and plasma treated (**b**) seeds of Zhongshuang 7 and non-treated (**c**) and plasma treated (**d**) seeds of Zhongshuang 11.

The apparent contact angle is shown in Fig. 2. Compared with the non-treated seeds, cold plasma treatment significantly decreased the apparent contact angle by 30.38% (*p* = 0.012) in Zhongshuang 7, and 16.91% (*p* = 0.024) in Zhongshuang 11, respectively.

### SOD activity

**Figure 3:**
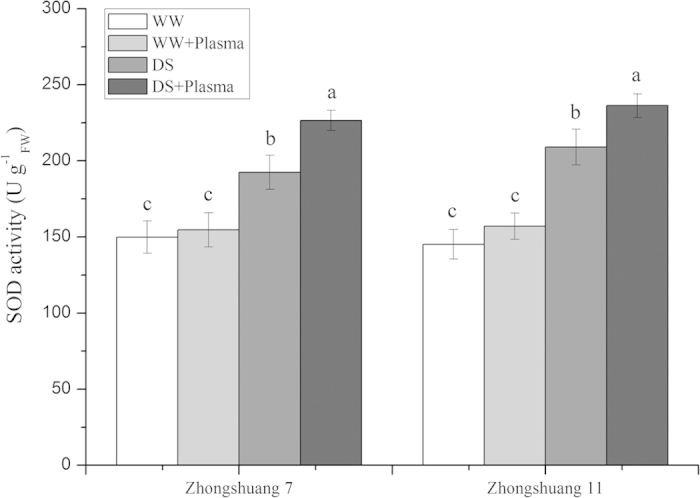
Effect of drought stress and cold plasma treatment on SOD activity in oilseed rape seedlings. WW: well watered; WW + Plasma: well watered + plasma; DS: drought stress; DS + Plasma: drought stress + plasma. Error bars indicate standard error (n = 3). Different lowercase letters denote statistical differences between treatment groups at the 5% level according to Duncan’s test.

SOD activities in the seedlings of Zhongshuang 7 and Zhongshuang 11 were markedly raised in all treatments under drought stress (Fig. 3). Cold plasma treatment significantly increased the SOD activity by 17.71% (*p* = 0.049) in Zhongshuang 7 and 13.00% (*p* = 0.025) in Zhongshuang 11, respectively, compared to the drought-stressed seedlings.

### CAT activity

**Figure 4:**
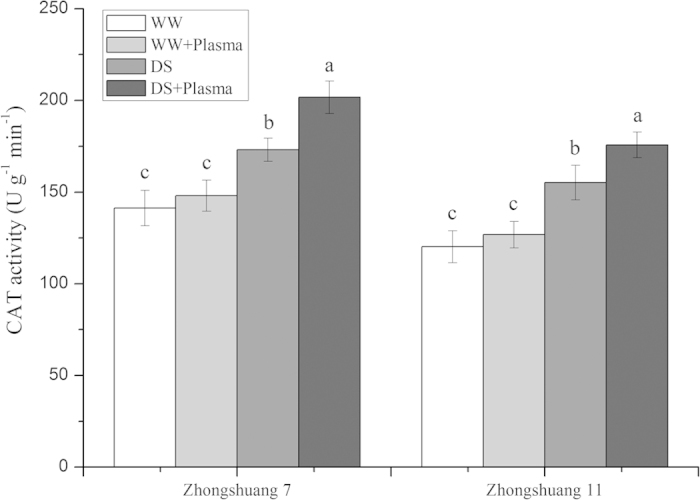
Effect of drought stress and cold plasma treatment on CAT activity in oilseed rape seedlings. WW: well watered; WW + Plasma: well watered + plasma; DS: drought stress; DS + Plasma: drought stress + plasma. Error bars indicate standard error (n = 3). Different lowercase letters denote statistical differences between treatment groups at the 5% level according to Duncan’s test.

Effect of drought stress and cold plasma treatment on the CAT activity is shown in Fig. 4. The CAT activity of Zhongshuang 7 and Zhongshuang 11 increased rapidly under drought stress. The cold plasma treatment improved the CAT activity by 16.52% (*p* = 0.033) in Zhongshuang 7 and 13.21% (*p* = 0.046) in Zhongshuang 11, respectively, compared to the drought-stressed seedlings.

### MDA content

**Figure 5:**
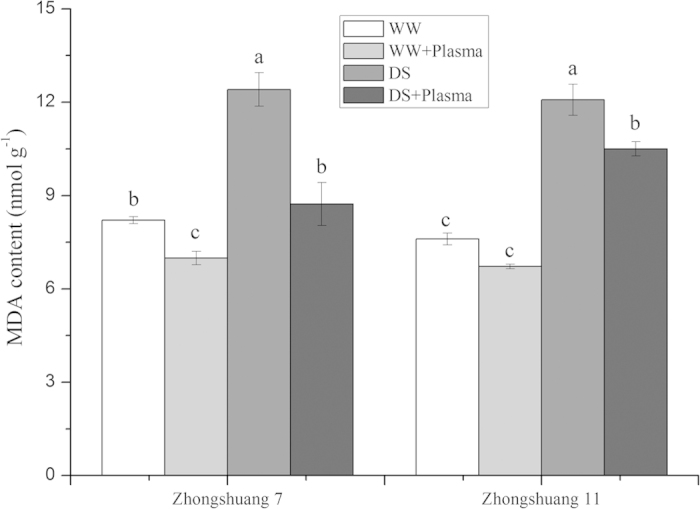
Effect of drought stress and cold plasma treatment on MDA contents in oilseed rape seedlings. WW: well watered; WW + Plasma: well watered + plasma; DS: drought stress; DS + Plasma: drought stress + plasma. Error bars indicate standard error (n = 3). Different lowercase letters denote statistical differences between treatment groups at the 5% level according to Duncan’s test.

MDA contents in the seedlings of Zhongshuang 7 and Zhongshuang 11 were markedly increased under drought stress (Fig. 5). The MDA content in plasma-treated Zhongshuang 7 was significantly reduced by 28.85% (*p* = 0.003), and that was reduced by 13.08% (*p* = 0.046) in Zhongshuang 11, respectively, compared with those of drought-stressed seedlings. The reduction in Zhongshuang 7 seedling MDA content after pre-treatment with cold plasma was significantly higher than that of Zhongshuang 11.

### Soluble sugar content

**Figure 6:**
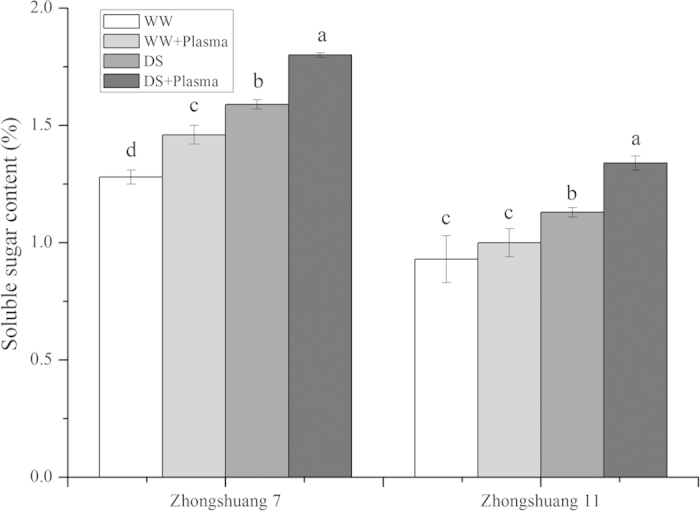
Effect of drought stress and cold plasma treatment on soluble sugar contents in oilseed rape seedlings. WW: well watered; WW + Plasma: well watered + plasma; DS: drought stress; DS + Plasma: drought stress + plasma. Error bars indicate standard error (n = 3). Different lowercase letters denote statistical differences between treatment groups at the 5% level according to Duncan’s test.

Soluble sugar contents in the seedlings of both cultivars were significantly increased in all treatments under drought stress (Fig. 6). Cold plasma treatment markedly increased the soluble sugar content in Zhongshuang 7, but had no obvious influence on Zhongshuang 11, under well watered conditions. After pre-treated with cold plasma, the soluble sugar content was significantly raised by 13.21% (*p* = 0.002) in Zhongshuang 7, while that was raised by 18.58% (*p* = 0.001) in Zhongshuang 11, respectively, compared with the drought-stressed seedlings.

### Soluble protein content

**Figure 7:**
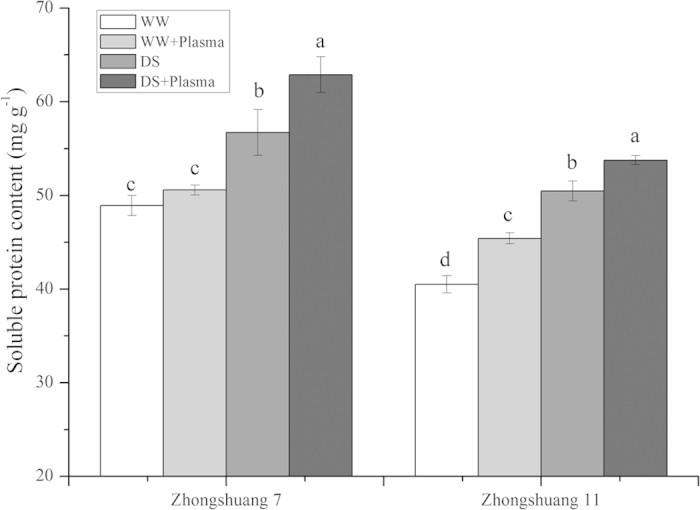
Effect of drought stress and cold plasma treatment on soluble protein contents in oilseed rape seedlings. WW: well watered; WW + Plasma: well watered + plasma; DS: drought stress; DS + Plasma: drought stress + plasma. Error bars indicate standard error (n = 3). Different lowercase letters denote statistical differences between treatment groups at the 5% level according to Duncan’s test.

Effect of the plasma treatment on soluble protein content was similar to its effect on soluble sugar content (Fig. 7). The soluble protein contents in the seedlings of both cultivars were markedly raised in all treatments under drought stress. Zhongshuang 7 showed greater improvement in soluble protein content than Zhongshuang 11. After pre-treated with cold plasma, the soluble protein contents in Zhongshuang 7 and Zhongshuang 11 were significantly increased by 10.90% (*p* = 0.034) and 6.04% (*p* = 0.046), respectively, compared to the drought-stressed seedlings.

## Discussion

In the present study, seed germination of oilseed rape was adversely affected by drought stress, but this was significantly ameliorated by the cold plasma treatment, especially for the drought-sensitive cultivar, Zhongshuang 7 (Table 1). This result is consistent with the findings of Selcuk *et al.* who found that plasma treatment significantly increased tomato seed germination^27^. Šerá *et al.* showed that poppy seed germination was also significantly increased by cold plasma treatment^28^. Richards’ function was used to fit the germination process of seed^29^,^30^. In our study, germination of oilseed rape seed was well followed to the Richards’ function (Fig. 1), the speed of germination in Zhongshuang 7 and Zhongshuang 11 were reduced by drought stress, and those were effectively promoted by the cold plasma treatment (Table 2). It is thought that cold plasma treatment improves absorptive ability, which might contribute to increased seed imbibitions, and therefore germination under drought stress^31^,^32^.

It is known that the growth status of plants affects their resistance to stress because improved growth can enhance plant resistance to stress^33^. Previous studies have suggested that plasma treatment significantly promotes poppy^28^, and tomato seedling growth^34^. This study has demonstrated that cold plasma treatment markedly increased seedling growth indices, i.e. shoot and root dry weights, shoot and root lengths, and lateral root number, which were significantly reduced by drought stress. Furthermore, cold plasma pre-treatment effect was pronounced on the drought-sensitive cultivar than on the drought-tolerant cultivar (Table 3). A study has shown that the cold plasma treatment significantly increased the dry weight of tomatoes under disease stress^26^. Plasma treatment also improved the growth of maize seedlings under cold stress^35^. Our results indicated that the improvement in resistance to drought stress in the oilseed rape seedlings pre-treated with cold plasma was partly due to their improved growth.

There is a direct association between wettability and germination of seed. The wettability of seed can be reflected by apparent contact angle, and with strong wettability seeds can absorb more water to stimulate seed germination under drought stress^30^,^36^. In the present study, cold plasma treatment significantly decreased the apparent contact angle of oilseed rape seed (Fig. 2). In agreement with this result, Li *et al.* who demonstrated that cold plasma treatment decreased the apparent contact angle of soybean seed^19^. Bormashenko *et al.* also found that cold radiofrequency air plasma treatment decreased the apparent contact angle of lentil and wheat seeds^32^. This suggested that the wettability of seed was improved by cold plasma treatment, which was important to improve seed germination by increasing water uptake of seed under drought stress.

Plants have naturally developed a number of physiological and biochemical strategies to adapt to drought stress. Drought stress usually induces the accumulation of ROS, which cause oxidative damage to plants. In contrast, plants can raise the efficiency of the antioxidant system activity, which improves their resistance to drought stress by increasing the levels of antioxidant enzymes, such as SOD, POD and CAT, and non-enzymatic compounds^10^,^37^. Plasma treatment plays an important role in regulating water balance by modulating antioxidant enzymes^35^. Plasma treatment stimulated SOD and POD activity in tomato seedlings^23^,^26^. In our study, the cold plasma treatment significantly increased the SOD and CAT activities in oilseed rape seedlings under drought stress (Figs 3 and 4). This implicates that the cold plasma treatment plays an important role to reduce oxidative damage and helps to maintain normal physiological metabolic activities, leading to improved oilseed rape seedling growth under drought stress.

The overproduction of ROS, induced by drought stress, leads to oxidative stress damage to protein and membrane lipids^38^. MDA is the product of membrane peroxidation and has been used as a direct indicator of lipid peroxidation and membrane damage^39^,^40^. The present study demonstrated that under drought stress conditions, the cold plasma treatment reduced membrane lipid peroxidation damage by increasing antioxidant enzyme activities, thus significantly reducing the accumulation of MDA induced by drought stress. Moreover, the cold plasma treatment effect was more pronounced on the drought-sensitive cultivar (Zhongshuang 7) than on the drought-tolerant cultivar (Zhongshuang 11) (Fig. 5). This result agreed with Yin *et al.* who reported that plasma treatment reduced MDA content in tomato seedlings^34^. Our results suggested that the cold plasma treatment can partially increase oilseed rape seedling growth by preventing the oxidative damage generated by drought stress.

Soluble sugar and protein contents are strongly correlated to improved stress tolerance in plants. The accumulation of soluble sugars and soluble proteins maintains plant turgidity, enhances absorptive ability, and protects membranes and macromolecules during drought stress^41^,^42^. Li *et al.* reported that cold plasma treatment significantly increased the soluble sugar and protein contents in soybean seedlings^19^. Chen *et al.* showed that the soluble sugar and protein contents in brown rice also increased when it was treated with cold plasma^21^. Our data indicated that the cold plasma treatment significantly increased the accumulation of soluble sugars and proteins in oilseed rape seedlings under drought stress, and the increase was greater in Zhongshuang 7 than in Zhongshuang 11 (Figs 6 and 7). Under drought stress, cold plasma treatment might maintain a favorable potential gradient for water uptake into the seedlings and increase energy supply to the plant through the increased accumulation of soluble sugars and soluble proteins. Hence, the cold plasma treatment would have alleviated the negative effects of drought stress on oilseed rape seedling growth.

Finally, it is concluded that cold plasma treatments alleviate drought stress damage in oilseed rape. The seed germination and seedling growth improvement was due to the cold plasma treatment via improvement in wettability of seed, antioxidant enzymes activities, soluble sugar and protein contents, and reduced lipid peroxidation-linked membrane deterioration. Thus, cold plasma treatment can be used in an ameliorative way to protect the oilseed rape seedlings against the damage caused by drought stress. However, further studies are needed to investigate the effects of cold plasma on oilseed rape growth and yield under drought stress.

## Materials and Methods

### Experimental apparatus

**Figure 8:**
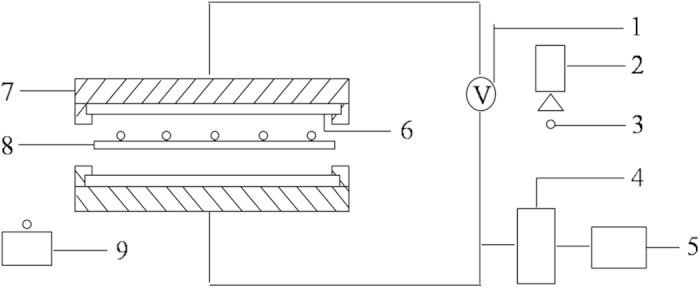
Experiment set-up of cold plasma. 1 radio frequency generator, 2 inlet hopper, 3 seeds, 4 cold trap, 5 vacuum pump, 6 plate, 7 metal suspension shell, 8 conveyer belt, 9 outlet hopper.

Experiment was performed in the commercial computer-controlled plasma treatment apparatus HD-2N, which was consisted of a vacuum device, cold plasma generator and transmission device and inlet/outlet hopper. The core of the cold plasma processing system is the cold plasma generator. The device is composed of two parallel plates and has a metal suspension shell. The area between the plates and the metal shell is filled with insulating materials. Seeds receive the non-ionizing radiation treatment when they are in the cavity between the two polar plates. Capacitive coupled plasma (CCP) was generated by radio frequency discharge. The apparatus is illustrated in Fig. 8.

### Treatment conditions

Healthy and uniform seeds were selected and exposed to inductive helium plasma discharge under the following parameters: the plasma frequency was 13.56 MHz, the power was 100 W, the pressure was 150 Pa and the volume of the discharge chamber was 1200 mm × 180 mm × 20 mm. The time span of irradiation was 15 s and the temperature of the discharge, measured by a thermistor, was about 25 °C. Meanwhile, control seeds were exposed to the same vacuum and helium flux as the treated seeds, but did not receive the plasma treatment. The above seedling measurements and content analyses were carried out approximately one day after the seeds had been treated with cold plasma.

### Plant material

Seeds of *Brassica napus* L. cv. Zhongshuang 7, a drought-sensitive cultivar and Zhongshuang 11, a drought-resistant cultivar were obtained from the Oil Crops Research Institute, Chinese Academy of Agricultural Science, Wuhan, China.

### Experimental design

The experiment was carried out at the Institute of Soil Science, Chinese Academy of Sciences, Nanjing, China (118°46′E, 32°03′N), between September, 2012 and December, 2013. Water limitation was applied using 15% (w/v) PEG 6000 (polyethylene glycol, Sigma). There were four treatments: (1) WW – well watered; (2) WW + Plasma – well watered + plasma; (3) DS – drought stress (15% PEG); (4) DS + Plasma – drought stress + plasma. The seeds were germinated in 9 cm petri dishes on two layers of filter paper. A total of 100 seeds were placed on each filter paper, and moistened with either 10 ml of PEG solution or 10 ml distilled water for the DS and WW treatments, respectively. The filter papers were replaced every 2 days to keep them completely moist. The seeds were incubated in a 25 °C light incubator. The seeds were considered to be germinated when the radicals were half the seed length. The germination percentage was recorded every 24 h for 7 days. Root length, shoot length, SOD and CAT activities, and soluble sugar and protein contents were measured on the 7^th^ day after germination. Seedlings were separated into shoots and roots for dry weight measurement. The experiment was a completely randomized design with three replicates.


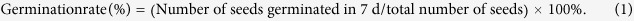



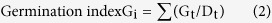


where G_t_ represents the number of germinated seeds on t day, and D_t_ represents germination days.


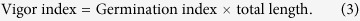


The ratio cumulative germination was calculated as in Šerá *et al.*^43^. The Richards’ function and population parameters *Vi* - final germination rate, index of viability; *Me* - median germination time, demonstrate the germination time of seed; *Qu* - dispersion, uniformity of seedling growth; and *Sk* - skewness, the composition of a population, were calculated according to Hara^44^.

The Richards’ function *Y*_*t*_ was calculated according to the following equation:


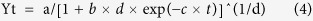


where *a*, *b*, *c* and *d* are the fitting parameters, and *t* is the time

### Determination of seed apparent contact angle

The apparent contact angle was established using a Kino goniometer (model SL200B).

### Determination of SOD and CAT activity

SOD activity was assayed according to the nitroblue tetrazolium method, and CAT activity was determined by monitoring the enzyme-catalyzed decomposition of hydrogen peroxide (H_2_O_2_) by potassium permanganate^45^.

### Determination of MDA, soluble sugar and protein contents

The MDA content was determined according to Health *et al.*^46^. The soluble sugar content was determined by the phenol sulfuric acid method^47^, and the soluble protein content was assayed using Bradford’s method^48^.

### Statistical analysis

All data are presented as the mean value ± standard error (SE) of three replicates. Analyses were performed using the SPSS statistical software package (Version 16.0) and the variance (*p* < 0.05) of the data was analyzed by one-way ANOVA (Duncan’s test).

## Additional Information

**How to cite this article**: Ling, L. *et al.* Cold plasma treatment enhances oilseed rape seed germination under drought stress. *Sci. Rep.* **5**, 13033; doi: 10.1038/srep13033 (2015).

## Figures and Tables

**Table 1:** Effect of drought stress and cold plasma treatment on seed germination of oilseed rape.

**Cultivar**	**Treatment**	**Germination rate (%)**	**Germination index**	**Vigor index**
Zhongshuang 7	WW	93.00 ± 0.58b	82.11 ± 0.57b	777.47 ± 16.82b
	WW + Plasma	99.70 ± 0.33a	88.67 ± 0.42a	942.97 ± 20.86a
	DS	80.00 ± 1.00d	59.00 ± 0.47d	544.90 ± 12.95d
	DS + Plasma	85.00 ± 0.48c	63.06 ± 0.38c	706.12 ± 6.36c
Zhongshuang 11	WW	98.00 ± 0.70a	89.89 ± 0.23a	662.24 ± 17.94c
	WW + Plasma	99.00 ± 0.58a	91.17 ± 0.50a	917.63 ± 40.08a
	DS	90.00 ± 1.53c	68.56 ± 1.23c	630.42 ± 15.42c
	DS + Plasma	94.00 ± 0.58b	71.83 ± 0.49b	799.87 ± 15.52b

WW: well watered; WW + Plasma: well watered + plasma; DS: drought stress; DS + Plasma: drought stress + plasma. Data were means ± SE of three replications. Different lowercase letters denote statistical differences between treatments groups at the 5% level according to Duncan’s test.

**Table 2:** Population parameters *Vi* (viability), *Me* (median germination time), *Qu* (dispersion) and *Sk* (skewness) of the Richards’ function for germination of oilseed rape.

**Cultivar**	**Treatment**	***Vi*** **(%)**	***Me*** **(d)**	***Qu*** **(d)**	***Sk*** **(%)**
Zhongshuang 7	WW	100.09 ± 0.01	1.04 ± 0.01	0.31 ± 0.01	0.41 ± 0.01
	WW + Plasma	100.10 ± 0.02	1.02 ± 0.01	0.32 ± 0.01	0.44 ± 0.01
	DS	100.18 ± 0.05	1.08 ± 0.01	0.28 ± 0.02	0.39 ± 0.02
	DS + Plasma	100.08 ± 0.02	1.03 ± 0.02	0.31 ± 0.01	0.41 ± 0.01
Zhongshuang 11	WW	100.04 ± 0.02	1.04 ± 0.01	0.27 ± 0.02	0.38 ± 0.01
	WW + Plasma	100.06 ± 0.02	1.05 ± 0.01	0.30 ± 0.01	0.39 ± 0.01
	DS	100.23 ± 0.06	1.10 ± 0.02	0.37 ± 0.01	0.44 ± 0.01
	DS + Plasma	100.09 ± 0.03	1.03 ± 0.01	0.34 ± 0.02	0.42 ± 0.02

WW: well watered; WW + Plasma: well watered + plasma; DS: drought stress; DS + Plasma: drought stress + plasma. Data were means ± SE of three replications.

**Table 3:** Effect of drought stress and cold plasma treatment on seedling growth of oilseed rape.

**Cultivar**	**Treatment**	**Shoot dry weight (mg•plant**^**−1**^)	**Root dry weight (mg•plant**^**−1**^)	**Shoot length (cm)**	**Root length (cm)**	**Lateral root number**
Zhongshuang 7	WW	2.47 ± 0.12ab	4.67 ± 0.24b	2.50 ± 0.03b	7.00 ± 0.11c	10.67 ± 0.30b
	WW + Plasma	2.70 ± 0.06a	5.78 ± 0.07a	2.93 ± 0.07a	7.70 ± 0.20b	14.00 ± 0.61a
	DS	1.80 ± 0.06c	2.77 ± 0.24d	1.03 ± 0.03d	8.17 ± 0.20b	8.00 ± 0.62c
	DS + Plasma	2.10 ± 0.05b	3.33 ± 0.12c	1.47 ± 0.09c	9.73 ± 0.20a	10.33 ± 0.34b
Zhongshuang 11	WW	2.70 ± 0.11b	4.50 ± 0.15b	2. 10 ± 0.06b	5.27 ± 0.14d	8.33 ± 0.30bc
	WW + Plasma	3.03 ± 0.07a	5.03 ± 0.23a	3.00 ± 0.10a	7.07 ± 0.30c	12.00 ± 0.59a
	DS	2.00 ± 0.11d	3.10 ± 0.09d	1.13 ± 0.03d	8.07 ± 0.20b	7.00 ± 0.62c
	DS + Plasma	2.30 ± 0.06c	3.57 ± 0.12c	1.47 ± 0.03c	9.67 ± 0.30a	9.67 ± 0.30b

WW: well watered; WW + Plasma: well watered + plasma; DS: drought stress; DS + Plasma: drought stress + plasma. Data were means ± SE of three replications. Different lowercase letters denote statistical differences between treatments groups at the 5% level according to Duncan’s test.
